# Non-biological methods for phosphorus and nitrogen removal from wastewater: A gap analysis of reinvented-toilet technologies with respect to ISO 30500

**DOI:** 10.12688/gatesopenres.12931.2

**Published:** 2020-05-15

**Authors:** Lena Trotochaud, Brian T. Hawkins, Brian R. Stoner

**Affiliations:** 1 Center for WaSH-AID, Duke University, Durham, NC, 27701, USA

**Keywords:** non-sewered sanitation systems (NSSS), reinvented toilet (RT), nutrient removal, nitrogen, phosphorus, ion exchange, membrane separation, hydrogels

## Abstract

The aims of the Reinvent the Toilet Challenge (RTTC) include creation of an off-the-grid sanitation system with operating costs of less than US$0.05 per user per day. Because of the small scale at which many reinvented toilets (RT) are intended to operate, non-biological treatment has been generally favored. The RTTC has already instigated notable technological advances in non-sewered sanitation systems (NSSS). However, increasingly stringent liquid effluent standards for N and P could limit the deployment of current RT in real-world scenarios, despite the urgent need for these systems. The newly adopted ISO 30500 standards for water reuse in NSSS dictate minimal use of chemical/biological additives, while at the same time requiring a 70% and 80% reduction in total nitrogen and phosphorus, respectively. This document provides a brief overview of the mature and emerging technologies for N and P (specifically ammonia/ammonium and orthophosphate) removal from wastewater. At present, the dearth of nutrient removal methods proven to be effective at small scales is a significant barrier to meeting ISO 30500 standards. Closing the gap between RTs and ISO 30500 will require significant investments in basic R&D of emerging technologies for non-biological N and P remediation and/or increased reliance on biological processes. Adaptation of existing nutrient-removal technologies to small-scale NSSS is a viable option that merits additional investigation.

## Introduction and scope

Anthropogenic nutrient pollution of surface waters has severe negative impacts on the environment, human health, and the economy. In particular, algal blooms, caused by eutrophication due to high levels of nitrogen and phosphorus (N and P, primarily as ammonia/ammonium and orthophosphate), pose immediate risks to wildlife and human populations, and it can cost millions of dollars to treat a single body of water once a bloom occurs ^1^. Several effective, mature technologies for mitigating environmental release of N and P are widely employed at municipal wastewater treatment plants (WWTPs). However, a technological gap exists in providing similarly effective nutrient removal at smaller, decentralized treatment systems while maintaining affordability and sustainability ^2^. While the cost, efficacy, and practicality of nutrient removal technologies depends on many factors unique to each wastewater stream, including the influent quality, effluent requirements, climate, land and capital availability, local cultural and institutional attitudes, etc ^3, 4^, a few general trends are observed ^1, 2, 5– 8^:

- The smaller the scale of the water treatment system, the higher the per capita cost.
- More stringent effluent standards for N and P require increasingly complex systems with multiple remediation processes (both biological and physical/chemical) working in concert.
- Biological methods of N and P removal typically have lower capital and operational/maintenance costs than chemical/physical methods.

The Bill & Melinda Gates Foundation’s Reinvent the Toilet Challenge (RTTC) has spurred significant advances in non-sewered sanitation systems (NSSS) designed to operate at less than US$0.05 per user per day with minimal use of chemical/biological additives. Typically designed to work at the household or institutional scale, these NSSS fall into the category of small-scale, decentralized treatment systems. Smaller-scale treatment systems which rely on biological processes are more susceptible to system shocks and stresses, including non-continuous/inconsistent influent supply/quality ^3, 9, 10^. Thus, many NSSS in the RTTC portfolio rely on non-biological ^†^ processes for water treatment and reuse (e.g. electrochemical oxidation; membrane separation) ^11– 18^. Treatment of the liquid fraction of NSSS waste to enable non-potable water reuse (e.g. for toilet flushing) has been a focus of many reinvented toilet (RT) technologies and guides the scope of this Open Letter.

The technological approach for N and P removal from liquid effluent will depend to a great extent on whether, how, and when urine and feces are separated from each other. Also important is whether and to what extent urine is diluted by water used for toilet flushing (and personal washing, where applicable). Urine contains approximately 90% of the total N and 50–65% of the total P in human excreta, and the chemical components are largely inorganic compounds (after urea hydrolysis); in contrast, the contributions to N and P from feces are largely organic (proteins and bacterial biomass) ^19^. For the purposes of this Open Letter, we focus on removal of ammonia/ammonium and orthophosphate, which are the majority water-soluble contributors to N and P in human excreta and the compounds of primary concern for anthropogenic nutrient pollution.

An additional layer of complexity has been introduced by the adoption of the new ISO 30500 standards for NSSS. The ISO 30500 standards provide guidance for safe onsite treatment of human excreta and non-potable water reuse, and includes threshold performance metrics for liquid effluent quality, including chemical oxygen demand (COD), total suspended solids (TSS), nutrients (N and P), and specific pathogens. Regardless of whether water is to be reused or discharged, ISO 30500 requires 70% and 80% reductions in total N and total P, respectively, in NSSS liquid effluent. Here, we briefly outline the current mature and emerging non-biological technologies for N and P removal. We discuss the advantages and limitations of these methods in the context of RTTC and their ability (or lack thereof) to reliably meet the ISO 30500 standards for nutrient removal. This discussion shows that, in general, the best conventional methods of N and P removal have not been shown to be compatible with small-scale RT systems. Throughout, we also provide perspective on current areas of research and development (R&D) opportunities that could spawn technological solutions which unify the goals of RTTC and ISO 30500. Finally, we briefly present potential opportunities for increasing the use of biological systems in the RTTC portfolio to meet more stringent effluent standards.

### Mature and emerging technologies for non-biological nutrient removal

Several non-biological technologies are commonly applied at municipal- and industrial-scale WWTPs for N and P removal. However, very few of these technologies have been employed at the household/community scale where NSSS are most relevant. Some methods (e.g. air stripping; breakpoint chlorination; chemical precipitation) are well-established but may be applicable only to one specific target nutrient, while others (e.g. hydrogel/polymer matrix encapsulation; ion-exchange materials; membrane-based separations) are in various stages of development and use depending on the nutrient targeted for removal/recovery. In this section, we give a brief overview of these mature and emerging technologies for N and P removal, while providing commentary on the relevant benefits and knowledge gaps as relates to their application in NSSS. A summary of the limitations and potential R&D opportunities for these technologies is presented in Table 1. It is important to note that the ISO 30500 standard is written with specific guidance (including liquid effluent reduction threshold values) for *removal* of N and P. While *recovery* and *reuse* of nutrients are critical topics for sustainability and implementation of NSSS technologies, the primary technological hurdle currently facing RT system deployment and water reuse is nutrient *removal*. We briefly mention situations where recovery/reuse is possible and indicate this also in Table 1, as we feel this is an important and exciting area for continued research. However, a comprehensive review of nutrient recovery/reuse is outside the scope of this Open Letter.

**Table 1.** Non-biological methods of N and P removal. Values in italic, lowercase text indicate potential technological limitations for use in NSSS. Values in all capital letters indicate potential technological advantages for use in NSSS. Values in bold text indicate areas of existing R&D opportunity.

Method	Nutrient removed (N, P, or both)	Requirements/restrictions (YES/NO)						Notes
		Chemical additives?	Other consumables?	Power consumption?	Component fouling?	Regeneration possible?	Resource recovery possible?	
Air stripping	N (as NH _3_)	yes*	NO	*yes*	NO	n/a	YES ^#^	*Large quantities of base required to raise pH. ^#^Typically requires precipitation in sulfuric acid.
Breakpoint chlorination	N (as N _2_)	yes*	*yes* ^#^	*yes*	*yes* ^#^	*no* ^#^	*no*	*Salt addition may be required depending on effluent quality. ^#^Costly noble-metal electrodes are eventually poisoned.
Chemical precipitation	P (using Al, Fe, Ca) both (using Mg)	*yes*	*yes*	NO	NO	*no*	YES*	*Cost and processes not optimized.
Hydrogel/polymer matrix encapsulation	both	NO	yes*	NO	Unknown **^#^**	**Unknown ^#^**	**Unknown ^#^**	*Assumes hydrogel cannot be regenerated. ^#^These technologies are not mature – requires additional R&D.
Ion-exchange materials	both	NO	yes*	NO	**Unknown ^#^**	**YES ^#^**	**YES ^#^**	*Adsorbent must be replaced or regenerated. ^#^R&D opportunities in materials discovery, surface chemistry modification, and resource recovery.
Membrane-based separations	both	NO	yes*	*yes*	***yes*^#^**	**Unknown ^#^**	**YES**	*Assumes membrane has a finite lifetime and cannot be regenerated. ^#^Membrane technologies are not mature – requires additional R&D.

### Air stripping

Air stripping is a process which relies on the aqueous equilibrium between the ammonium ion (NH _4_^+^) and free ammonia (NH _3_). Under optimal conditions, this equilibrium is shifted to favor free ammonia which can leave the solution via evaporation. As a physical process, air stripping efficiency is affected by the ambient temperature and pressure. However, the solution pH is the predominant factor in determining availability of free NH _3_; higher pH heavily favors NH _3_, with the optimal pH for air stripping being ≥10. Optimized NH _3_ removal via air stripping is in excess of 90% ^3, 20, 21^.

Chemical addition of large quantities of base is required to bring the wastewater pH into the optimal range for air stripping. It follows that acid addition is often required to bring the pH back down to the acceptable range for subsequent effluent discharge. Significant investments in infrastructure and/or land can be required for stripping towers, aeration ponds, etc ^20^. While it may be possible to effectively scale down the infrastructure, conventional ammonia air stripping is largely incompatible with RTTC guidelines due to the need for caustic chemical additives.

Electrochemical stripping may be one viable alternative to conventional air stripping for decentralized systems where intermittent electricity or photovoltaics are available ^22^. In electrochemical stripping, base production is accomplished *in situ* using electricity and an appropriate anode material, and ammonia is separated from the solution via a gas-permeable membrane and without the need for a stripping tower. This emerging technology faces several challenges, including electrode poisoning/stability, membrane fouling, and generation of undesirable byproducts. These issues will be discussed in more detail in the subsections below.

### Breakpoint chlorination

For ammonia-containing wastewater, breakpoint chlorination describes the chemical process whereby a sufficient amount of hypochlorous acid is present to completely oxidize ammonia to nitrogen gas:

$${2\text{NH}}_{\text{4}}^{+}+\;3\text{HOCl}\;\to \;{\text{N}}_{2}+\;3{\text{H}}_{2}\text{O}\;+\;3\text{HCl}\;+\;2{\text{H}}^{+}$$

The above description and equation are deceptively simplified, as multiple intermediate steps involving the formation and subsequent reaction of chloramines are not explicitly shown. Rather than adding chlorine directly to wastewater, hypochlorous acid can be generated *in situ* electrochemically by catalytic oxidation of chloride ions at an appropriate electrode surface. Breakpoint chlorination can reliably give ammonia-to-nitrogen conversions of ≥95% with no measurable nitrous oxide formation under appropriate operating conditions ^21, 23^

Breakpoint chlorination initially appears to be an attractive method of N removal, as it typically requires no chemical additives (unless the chloride concentration in the wastewater influent is too low, in which case salt, e.g. sodium chloride, addition is warranted), generates harmless nitrogen gas, and uses technology already deployed in many NSSS. (Electrochemical oxidation of chloride for liquid disinfection is currently used in several RT systems ^14– 16^.) However, there are some important limitations, including the possibility for formation of undesirable oxidation byproducts, many of which are toxic or carcinogenic ^24– 26^. The formation of undesired byproducts has been well documented for electrochemical wastewater treatment processes, including chlorine disinfection ^20, 27, 28^. Breakpoint chlorination for ammonia removal uses the same active compounds as chlorine disinfection, but at far higher concentrations, which dramatically increases the chances for formation of harmful byproducts. In fact, the chloramine reaction intermediates themselves are considered undesired byproducts ^3, 20, 21^. This makes incomplete oxidation of ammonia to nitrogen problematic, which drives demand for an excess of reactive free chlorine, and necessitates a downstream dechlorination process. Control and remediation of byproducts downstream of the breakpoint chlorination process is thus required, which adds complexity and cost ^3, 20, 27^.

Additional issues with breakpoint chlorination include: (1) acidification of the treated effluent and subsequent creation of nitrogen trichloride; (2) need for chemical addition to neutralize pH, causing large increases in total dissolved solids in the treated effluent; (3) finite lifetimes of electrode materials (which contain expensive noble metals and metal oxides) due to surface inactivation/poisoning; (4) need for automated monitoring and control of pH, ammonia, and free chlorine levels; (5) practicality primarily as a polishing technique, rather than for removing high levels of N; (6) does not allow for recovery of N in a bioavailable form ^3, 20, 21^. Despite the reliability and efficiency of breakpoint chlorination, the associated downstream problems should exclude its use in the RTTC portfolio. There may be other electrochemical processes for N removal that are less problematic and more effective, e.g. electrodialysis, which will be discussed in more detail below.

### Chemical precipitation

Chemical precipitation involves the addition of soluble salts to nutrient-rich wastewater to induce formation of nutrient-containing, insoluble compounds, which are removed by settling (gravity) or filtration. Chemical precipitation is currently the primary method of P remediation in wastewater treatment applications, and the most common compounds used for P removal contain Fe, Al, Ca, and/or Mg ^29^. Aside from the obvious requirement for chemical additives, a potential drawback of chemical precipitation is that P and/or N is merely sequestered rather than being removed from the wastewater treatment system. A consequence of this is the generation of large quantities of sludge that still require disposal and/or subsequent treatment. The sludge quantity is further increased by the need for chemical additives in excess of the theoretical stoichiometric requirements, presumably due to various side reactions ^2, 20^. Small-scale NSSS may lack the space and/or infrastructure required to handle large amounts of sludge on site.

The particular chemical additive to be used in any treatment system needs to be chosen with care, as problems such as scaling and slow precipitation kinetics will be heavily influenced by the influent water chemistry. Often, pH adjustment by addition of hydroxide salts is required to favor formation of the desired precipitate ^20^. The choice of cation used for precipitation is critical and affects myriad factors including overall cost, P removal efficacy, optimal pH and temperature required for P removal, and reusability/bioavailability of the P-containing precipitate ^30^. Resulting trade-offs need to be carefully considered depending on the context and effluent requirements. For example, the Fe and Al salts used for P precipitation are typically much less expensive than Ca and Mg salts; however, Fe and Al phosphate precipitates are not suitable for direct use as fertilizers due to low P bioavailability ^31^. On the other hand, using Mg for struvite precipitation can enable simultaneous removal of phosphate and ammonium in a 1:1 stoichiometric ratio and has been shown to be widely bioavailable; ^30^ however, struvite precipitation has a narrow window of optimal pH and cannot be relied upon as the sole method of N removal due to the much higher concentrations of ammonia/ammonium relative to phosphate ^32^. Furthermore, more research is required to address concerns regarding the presence of contaminants in precipitates where fertilizer use is intended ^30– 32^.

There still exists some room for improvement of chemical precipitation technologies, e.g. with the novel application of materials such as calcium silicate hydrate, which can initiate precipitation and simultaneously adjust pH ^33^, thus decreasing the quantity of added chemicals needed for phosphate removal. Electrochemically-induced coagulation or precipitation using sacrificial anodes can provide better control of critical process parameters, including pH and metal ion dosing, and may be easier to implement than direct salt addition in decentralized treatment settings ^– 36^. If the nutrient-containing precipitate is a valuable commodity (e.g. fertilizer) and can be efficiently separated from sludge, the use of a chemical additive may be justified by the benefits endowed from nutrient recovery. However, converting chemically-bonded nutrients in some precipitates to a bioavailable form can be difficult ^2^. New nanocomposite materials could be designed to increase capacity and regeneration capability ^37^. Depending on the influent characteristics, some industrial byproducts (e.g. gypsum, fly ash, slag) may provide a cost-effective solution for nutrient remediation ^29^. Ultimately, the overall cost, availability, sludge disposal, and potential downstream environmental impact of chemical precipitation processes will need to be evaluated on a case-by-case basis for each wastewater stream and its corresponding effluent requirements.

### Hydrogel/polymer matrix encapsulation

Hydrogels and polymer matrices are a relatively new class of low-cost adsorbent materials being studied for P and N capture ^38– 42^. Hydrogels have a high water content and porous structure that facilitates solute diffusion ^43^. As these materials are synthetic, they offer the opportunity to tune the absorptive selectivity and capacity by altering the polymer chemistry. Hybrid hydrogels can also be created by embedding inorganic particles into the polymer matrix, which can further increase the adsorption capacity ^43^. Hydrogels also have high potential for recycling and regeneration ^43, 44^. To date, research for wastewater treatment with hydrogels has primarily focused on adsorption of organic compounds, radionuclides, and dyes ^43, 44^. Recently, a bentonite-based hybrid hydrogel showed selectivity for phosphate adsorption in a mixed-anion waste stream with 99% phosphate removal under optimal conditions ^38^. A commercially available polymer hydrogel was demonstrated to have ammonia removal capacity up to 80% at pH 5.0–8.0, and showed minimal performance loss after regeneration with mild acid washing ^42^. Development of new, tailored hydrogels for P and N removal is a promising area for future research.

### Ion-exchange materials

Ion-exchange materials are highly porous structures loaded with ions which are selectively displaced by target ions (e.g. ammonium; phosphate) in the wastewater stream. Ion-exchange materials offer several benefits when compared to conventional chemical precipitation methods. For example, while chemical precipitation can handle higher nutrient concentrations, the kinetics of ion-exchange processes are much faster in comparison and thus the removal efficiency is not highly dependent on retention time. Additionally, nutrients captured by ion exchange are more easily recovered in comparison to e.g. chemical precipitation, as ion-exchange processes are reversible ^29^.

A fundamental limitation of ion-exchange materials is that their capacity is inherently limited by the accessible surface area and ion loading of the material; at some point, the adsorbent will need to be replaced or regenerated. Synthetic ion-exchange resins, while potentially engineered to have better selectivity, adsorption and regeneration capacities, and durability than natural zeolites, can be far more costly ^45^. On the other hand, certain natural zeolites may be geographically scarce. Conventional methods of material regeneration require large volumes of water and quantities of salts, including caustic acids and bases ^46^. There is also the issue of the so-called “paradox of P sorption materials”, wherein the materials that are highly effective at P removal generally show poor performance for water transport ^29^.

Despite these issues, there is opportunity for using ion-exchange materials in a RTTC context. In particular, the option to regenerate the adsorbent material and recover a nutrient-rich solution is attractive when considering strategies for integrating NSSS into a circular N and/or P economy. For any particular ion-exchange material, testing with the real, intended wastewater stream is critical, as influent properties such as pH and organic loading can have dramatic effects on adsorption and regeneration capacities ^9, 45^. Offsite zeolite regeneration using a spoke-hub model could create local employment opportunities, as the regeneration/recovery process need not be highly technical and the regeneration solution can be reused many times. However, access to and cost of salts required for regeneration would still need to be considered ^45^. Optimizing the zeolite regeneration process to operate on a small scale with limited additives would be a significant achievement that merits investigation ^46^.

Even without regeneration, the low cost of certain natural zeolites and other minerals may make them effective as consumable filtration media in small-scale systems. For example, clinoptilolite is one of the best natural ion exchange materials for ammonium ions ( capacity = 2–30 mg NH _4_^+^ g ^-1^), and has been demonstrated to have a lower material cost ($ per g of N removed) than conventional biological nitrogen removal, even when treated as a one-time-use consumable ^45^. Spent natural zeolites may have additional value as soil conditioners and fertilizers ^46– 48^. Polonite ^®^ is a particularly attractive material for P removal and recovery due to its high P capacity (up to 12% by weight) ^49– 51^, and reasonable price (< US$1 kg ^-1^). The exhausted filter media can be applied directly to soil for agricultural use as a slow-P-releasing fertilizer and soil conditioner ^52^. If the two processes are used in series, the more basic Polonite ^®^-filtered effluent could counteract the undesired pH drop induced by *in situ* chlorine disinfection. We are currently evaluating the use of Polonite ^®^ and clinoptilolite for ISO 30500 compliance in NSSS.

Ammonia removal from wastewater using natural ^53^ and synthetic ^9, 48^ ion-exchange materials has been studied for several decades; for the natural zeolite clinoptilolite, the average ammonia removal efficiency is ≥95% ^21^. Despite the use of ion-exchange materials for decades in remediation of ammonia, ion-exchange materials can still be considered an “emerging technology” for P removal in wastewater treatment applications. Hybrid anion-exchange resins (HAIX) consist of iron (hydr)oxide particles embedded within anion-exchange polymers and have been tested with a variety of wastewater streams, including source-separated urine ^54, 55^. The operational capacity of HAIX media depends on both the influent P concentration and the target effluent P concentration ^56, 55^, and thus use of HAIX in NSSS will need to be assessed on a case-by-case basis. More work is also needed to demonstrate the economic feasibility of these materials (including the regeneration process) for use in small-scale NSSS; field testing and pilot-scale studies will be critical for evaluating long-term viability of HAIX. Overall, there are ample opportunities for fundamental R&D in synthetic ion exchange resins, as well as modified and unmodified natural zeolites, for nutrient removal in NSSS ^48, 57^.

### Membrane-based separations

Membrane-based separation technologies are emerging as promising methods of nutrient removal for small-scale water treatment systems. Both active (energy required to drive separation; e.g. electrodialysis) and passive (separation driven by chemical equilibrium; e.g. osmosis, ion-selective membranes) membrane separation methods are dynamic areas of research for nutrient removal applications. For example, forward osmosis from source separated urine was recently shown to give at least 50% N and 93% P recovery into a diluted fertilizer draw solution. However, typical issues of membrane scaling and fouling were found to increase the operating costs of this system, due to the need for membrane replacement ^58^.

All membrane-based technologies suffer from a few key limitations in the membranes themselves, including membrane fouling and poor selectivity ^59^. However, important fundamental advances are continually being made in the development of tailored membrane materials ^60^. For example, highly selective membranes have recently been fabricated using covalent organic frameworks with tunable functional groups and nanoporous structures ^60^. However, cost and scalability of any new membrane materials must be considered from the earliest stages of development for application in NSSS, particularly for deployment in low- and middle-income countries. Opportunities also exist in modification of existing membranes and processes to tailor them for use in NSSS. Membrane surface modification can be used to increase hydrophilicity and membrane selectivity. Surface modification was recently used to demonstrate >89% ammonium removal in a non-optimized forward osmosis system ^61^. Optimization of operating conditions and in depth techno-economic analyses of nutrient recovery may make membrane-separation-based technologies viable options under certain conditions. In particular, membrane fouling can be largely mitigated when membranes are used in “tertiary” nutrient removal processes (i.e. after COD removal).

Another promising method for mitigating membrane fouling is through electrodialysis reversal (EDR). In this process, the electrical polarity is switched periodically between the anode and cathode material to enable *in situ* self-cleaning of the electrode and membrane surfaces ^59^. EDR is already widely used in water desalination technologies, and one company (Saltworks Technologies Inc.) has developed a modular EDR cell stack demonstrating >95% ammonia removal in a municipal WWTP. Whether electrodialysis can be cost effective on a small scale and effectively handle more concentrated wastewater (blackwater) for use in NSSS will need to be demonstrated.

## Reconsidering biological N and P remediation at small scales

There is likely not a “one-size-fits-all” solution to tackling the problem of nutrient removal at small scales ^2^. Above, we outlined some of the opportunities for R&D in physical/chemical methods of nutrient removal in the context of NSSS. At present, there is also a significant knowledge gap as to whether biological systems can provide efficient nutrient removal in NSSS. With the exception of constructed wetlands ^62^, there is very little data available at the household/community scale ^2^. Some of the major limitations for using biological systems in NSSS is their susceptibility to environmental stress, long start-up times, infrastructure requirements, and technical complexity. Performance of biological systems would be highly dependent on the local climate, seasonal variations, and ecosystem, and would require appropriate pilot tests in addition to laboratory efforts. Addressing these issues at small scales has not been adequately investigated, which presents opportunities for fundamental R&D that could support the goals of RTTC. Some opportunities for further investigation are briefly outlined below.

The lines between traditional “biological” treatment processes (i.e. systems intentionally inoculated with specific microbial strains) and truly “non-biological” treatment methods are likely already blurred in many RT systems due to the high microbial load of feces, which could yield formation of “native” biofilms on or within system components. The role of native biofilms in nutrient sequestration thus cannot be ignored ^33^, and the conditions which favor biological N and P sequestration in native biofilms could be optimized; more work is needed in this area, and would be specific to a given wastewater stream and treatment context ^2^. Gravity-driven membrane filtration systems have been reviewed recently and it is proposed that these systems can be economically viable at household- and community-scales ^63^. The formation of a stable biofilm on the membrane surface is a critical factor in their operation. The nutrient removal capabilities of these membrane systems have not been optimized for use in small-scale NSSS.

Hybrid chemical/biological systems are known to meet ISO 30500 standards for nutrient removal on large scales, but adapting these technologies to small scales will require extensive re-engineering and research efforts. For P removal in particular, it has been shown that it may not be possible to downscale existing technologies while maintaining performance ^2^. Nevertheless, some emerging technologies which combine chemical and biological processes show promise for efficient nutrient removal and recovery at small scales ^28, 64, 65^. Combining FeCl _3_ dosing with a membrane bioreactor and cofermentation was recently demonstrated, yielding >98% P removal and subsequent recovery (>60%) from the bioreactor sludge ^66^. (The precipitate recovered in this case was vivianite, which may have value in electronic applications and as a fertilizer precursor.) ^67, [Bibr ref-68]^ An electrochemically assisted, small-footprint, constructed wetland recently demonstrated >99% phosphate and >93% total nitrogen removal in tertiary wastewater treatment ^69^. Developing hybrid chemical/biological technologies specifically for small-scale blackwater treatment could yield breakthroughs in nutrient removal efficiencies.

The generation of biomass inherently consumes large quantities of N and P. The biomass product can serve as a valuable commodity, e.g., as an animal food supplement or via conversion to biofuel. One interesting area for future research would be in exploiting the eutrophication process by intentionally cultivating algae in a NSSS ^70, 71^. Algae can remove N and P from wastewater at least as effectively as many chemical treatments and can be a high-value biomass commodity ^2, 72^. As algae species are ubiquitous in aqueous environments, biological additives may not be necessary if the conditions for sustained algal growth are optimized, and nutrient removal efficiency may be independent of the particular algal community cultivated ^73^. Algae cultivated in a NSSS could be dried passively in the sun and used as a nutrient supplement for livestock in the local community. For example, adding small amount of dried algae to cows’ food can increase the omega-3 content of their milk ^74^ and decrease methane emissions ^75^. Engineering innovations are still needed to optimize small-scale algae cultivation, as well as algal biofilm stability, separation, and recovery ^2, 76– 78^. In particular, adapting algal-based systems to optimize light exposure in a minimal areal footprint would be critical for implementation in high population-density areas.

## Conclusion

Rather than seeking a silver-bullet solution, installing sustainable methods for small-scale, decentralized nutrient removal will necessitate a case-by-case approach that takes into account the myriad technical, cultural, and economic constraints unique to each water-use scenario. The need for customized sanitation solutions should motivate investment in basic R&D for emerging technologies, including non-biological, biological, and hybrid solutions. The development of reliable non-biological nutrient removal methods may be critical especially for use in colder climates, and there is ample research space to explore options including ion-exchange processes, hydrogel materials, and membrane-driven separations. Furthermore, the potential of biological solutions for nutrient removal has been underappreciated in the RT portfolio to date. Incorporation of biological components may be indispensable in meeting the ISO 30500 effluent requirements in certain situations.

## Data availability

No data is associated with this article.

## Notes

^†^Because of the nature of blackwater, it is inevitable that biological processes occur at some point in many (if not all) of the RTTC systems. Here, we use “non-biological” to distinguish methods where biological processes are not being intentionally targeted or optimized to function as components of the wastewater treatment process.
