# Supplementary material for: Non-biological methods for phosphorus and nitrogen removal from wastewater: A gap analysis of reinvented-toilet technologies with respect to ISO 30500
Source: Gates Open Res. 2020 May 15;3:559. Originally published 2019 Feb 24. [Version 2] doi: 10.12688/gatesopenres.12931.2 (PMC7232852; doi:10.12688/gatesopenres.12931.2)
Supplement: Peer review history

# Peer review: Reviewer response for version 2

**DOI:** 10.21956/gatesopenres.14324.r28852

**Published:** 2020-05-15

**Authors:** Lauren F. Greenlee

**Affiliations:** 1 Ralph E. Martin Department of Chemical Engineering, University of Arkansas, Fayetteville, AR, USA

No additional comments.

Does the article adequately reference differing views and opinions?

Partly

Are all factual statements correct, and are statements and arguments made adequately supported by citations?

Yes

Is the Open Letter written in accessible language?

Yes

Where applicable, are recommendations and next steps explained clearly for others to follow?

Partly

Is the rationale for the Open Letter provided in sufficient detail?

Yes

Reviewer Expertise:

Water treatment; electrochemical engineering; electrocatalysis; nutrient recovery; nanotechnology

I confirm that I have read this submission and believe that I have an appropriate level of expertise to confirm that it is of an acceptable scientific standard.

# Peer review: Reviewer response for version 1

**DOI:** 10.21956/gatesopenres.14033.r27001

**Published:** 2019-04-24

**Authors:** Lauren F. Greenlee

**Affiliations:** 1 Ralph E. Martin Department of Chemical Engineering, University of Arkansas, Fayetteville, AR, USA

The article presents a useful overview of N and P removal/recovery options from the perspective of small-scale RT systems.

 Specific comments:

1. The introduction mentions several times “mature” or “conventional” technology solutions for removing N and P. I think it would be helpful to have a brief description early on (maybe just a few words or specific examples) of what these technology solutions are so that the reader knows up front what these technologies are – it could make the transition from the introduction to the core of the article better.
2. Is there a large energy demand to air stripping, and/or for the other processes mentioned? How does the energy demand compare across the technology space and how are the energy supply challenges addressed for the small-scale RT systems?
3. I would recommend electrochemical N and P recovery be added to Table 1. See for example: Hug and Udert (2013[ref-1); Lin *et al.* (2018 ^2^); https://www.igb.fraunhofer.de/en/research/competences/physical-process-technology/nutrient-management/phosphorus-recovery.html.
4. Is there any movement in the hydrogel/polymer matrix or ion exchange resin research toward not just N and P removal but recovery as a usable composite for fertilization? For example, designing systems where the polymer or resin used could actually be directly land applied as a soil amendment? You have Polonite as an example, but are there other efforts in this area?
5. The sentence on the “paradox of P sorption materials” describes the materials that are good at P removal and are poor water conductors. I would recommend changing the wording to say “poor performance for water transport”. The word “conductor” implies conduction, which would be transport of heat/electrons, but I think you mean that the movement of water molecules through these materials is poor, which would be related to the convective/diffusive transport of water.
6. The authors mention new developments in the membrane design/materials field. It might be important to also comment on the cost and scalability of these new membrane designs, as these two factors are often extremely limiting for new membrane materials. For researchers in this area reading this article, it’s important for people to think about cost and scalability from the beginning when designing new materials for this application.

Does the article adequately reference differing views and opinions?

Partly

Are all factual statements correct, and are statements and arguments made adequately supported by citations?

Yes

Is the Open Letter written in accessible language?

Yes

Where applicable, are recommendations and next steps explained clearly for others to follow?

Partly

Is the rationale for the Open Letter provided in sufficient detail?

Yes

Reviewer Expertise:

Water treatment; electrochemical engineering; electrocatalysis; nutrient recovery; nanotechnology

I confirm that I have read this submission and believe that I have an appropriate level of expertise to confirm that it is of an acceptable scientific standard.

## References

[1] : Struvite precipitation from urine with electrochemical magnesium dosage. *Water Res*.2013;47(1) : 10.1016/j.watres.2012.09.036 289-99 10.1016/j.watres.2012.09.036 23123049

[2] : Struvite precipitation from biogas digestion slurry using a two-chamber electrolysis cell with a magnesium anode. *Journal of Cleaner Production*.2018;174: 10.1016/j.jclepro.2017.10.224 1598-1607 10.1016/j.jclepro.2017.10.224

# Response

**Published:** 2020-05-08

**Authors:** Lena Trotochaud

We sincerely thank the Reviewer for their insightful comments and suggestions. We have addressed each of their comments below, and we feel that the manuscript is much improved due to their feedback. The Reviewer's comments are copied below in *italic text*, and our responses are in plain text. Changes to the manuscript are shown in **bold text.**

*The article presents a useful overview of N and P removal/recovery options from the perspective of small-scale RT systems.*

 We sincerely thank the Reviewer for their comments and for the helpful suggestions for improvement.

*Specific comments:*

*1.  The introduction mentions several times “mature” or “conventional” technology solutions for removing N and P. I think it would be helpful to have a brief description early on (maybe just a few words or specific examples) of what these technology solutions are so that the reader knows up front what these technologies are – it could make the transition from the introduction to the core of the article better.*

 We have added more specific examples to the beginning of the section “Mature and emerging technologies for non-biological nutrient removal” to better integrate with and transition to the later subsections:

 “Some methods (e.g. air stripping **; breakpoint chlorination; chemical precipitation**) are well-established **but may be** applicable only to one specific target nutrient, while others (e.g. **hydrogel/polymer matrix encapsulation**; ion-exchange materials; **membrane-based separations**) are in various stages of development…”

*2.  Is there a large energy demand to air stripping, and/or for the other processes mentioned? How does the energy demand compare across the technology space and how are the energy supply challenges addressed for the small-scale RT systems?*

 We specifically included a column in Table 1 to acknowledge which technologies will require electricity from some source, however, it is difficult to put a number on energy demand for any given technology for use in small-scale NSSS when the only available data are for conventional systems at large wastewater treatment plants (e.g. air stripping). Furthermore, the energy demand of some technologies can fluctuate over time and during periods of intermittent use, which will be more noticeable in a small-scale system than for a large WWTP. In our experience, it is extremely difficult to estimate energy demand for small-scale NSSS without context-specific field/pilot testing and/or an in-depth techno-economic analysis.

 The original aim of the Reinvent the Toilet Challenge was to create a toilet that “operates ‘off the grid without connections to water, sewer, or electrical lines”. Strictly speaking, any RT system would ideally generate its own electricity (e.g. with thermoelectrics) or use solely renewable sources (e.g. solar) for all power needs. However in practice, some basic electrical connectivity is assumed in many RT prototype systems. Nevertheless, energy requirements are a critical consideration for RT systems, due to lack of reliability of grid power in many rural areas and some low- and middle-income countries, as well as the high cost of electricity in some areas, even when it is reliably available.

*3.  I would recommend electrochemical N and P recovery be added to Table 1. See for example: Hug and Udert (2013[ref-1); Lin et al. (2018* *^2^* *);* *https://www.igb.fraunhofer.de/en/research/competences/physical-process-technology/nutrient-management/phosphorus-recovery.html* *.*

 Electrochemically-induced coagulation or struvite precipitation is indeed an interesting area of research, with several benefits over salt addition, including better control of pH and metal ion dosing. However, the chemistry involved in formation of precipitates is identical, so we believe adding it as a separate item in the table is not necessary. However, we have added the following text and citations to acknowledge the benefits of electrochemically-induced coagulation/precipitation:

 “ **Electrochemically-induced coagulation or precipitation using sacrificial anodes can provide better control of critical process parameters, including pH and metal ion dosing, and may be easier to implement than direct salt addition in decentralized treatment settings.[Lacasa 2011, Hug 2013, Garcia-Segura 2017]**

 Lacasa E, Cañizares P, Sáez C, Fernández FJ, Rodrigo MA: Electrochemical phosphates removal using iron and aluminium electrodes. *Chem. Eng. J.* 2011;172(1):137-143

 Hug A, Udert KM: Struvite precipitation from urine with electrochemical magnesium dosage. *Water Res.* 2013;47(1):289-99. 10.1016/j.watres.2012.09.036

 Garcia-Segura S, Eiband MMSG, de Melo JV, Martínez-Huitle, CA: Electrocoagulation and advanced electrocoagulation processes: A general review about the fundamentals, emerging applications and its association with other technologies. *J. Electroanal. Chem.* 2017;801:267-99. 10.1016/j.jelechem.2017.07.047

*4.  Is there any movement in the hydrogel/polymer matrix or ion exchange resin research toward not just N and P removal but recovery as a usable composite for fertilization? For example, designing systems where the polymer or resin used could actually be directly land applied as a soil amendment? You have Polonite as an example, but are there other efforts in this area?*

 We are not aware of any examples of hydrogel/polymer or ion exchange resin development for direct application as fertilizer or soil amendment, although this would be an interesting area for future research. There are several examples of mineral/zeolite based materials, including Polonite and clinoptilolite which we mentioned. However, the general strategy and research focus with ion exchange and polymer-based materials seems to be to regenerate the material and use it repeatedly, and recover nutrients from the regeneration solution. Nevertheless, it seems that many of these materials cannot be regenerated indefinitely, which raises the question as to what to do with them when they have reached full exhaustion. While considering this comment, we realized that we neglected to mention the hybrid-anion exchange resins (HAIX) that have been tested recently for phosphate removal and recovery. We have added the following text and references to include these important materials:

 “ **Hybrid anion-exchange resins (HAIX) consist of iron (hydr)oxide particles embedded within anion-exchange polymers and have been tested with a variety of wastewater streams, including source-separated urine.[Sengupta 2011, O’Neal 2013] The operational capacity of HAIX media depends on both the influent P concentration and the target effluent P concentration,[Martin 2017, O’Neal 2013] and thus use of HAIX in NSSS will need to be assessed on a case-by-case basis. More work is also needed to demonstrate the economic feasibility of these materials (including the regeneration process) for use in small-scale NSSS; field testing and pilot-scale studies will be critical for evaluating long-term viability of HAIX.**”

 Sengupta S, Pandit A: Selective removal of phosphorus from wastewater combined with its recovery as a solid-phase fertilizer. *Water Res.* 2011;45(11):3318-30. 10.1016/j.watres.2011.03.044

 Martin BD, De Kock L, Gallot M, Guery E, Stanowski S, MacAdam J, McAdam EJ, Parsons SA, Jefferson B: Quantifying the performance of a hybrid anion exchanger/adsorbent for phosphorus removal using mass spectrometry coupled with batch kinetic trials. *Environ. Technol.* 2018;39(18):2304-14. 10.1080/09593330.2017.1354076

 O’Neal JA, Boyer TH: Phosphate recovery using hybrid anion exchange: Applications to source-separated urine and combined wastewater streams. *Water Res.* 2013;47(14):5003-17. 10.1016/j.watres.2013.05.037

*5.  The sentence on the “paradox of P sorption materials” describes the materials that are good at P removal and are poor water conductors. I would recommend changing the wording to say “poor performance for water transport”. The word “conductor” implies conduction, which would be transport of heat/electrons, but I think you mean that the movement of water molecules through these materials is poor, which would be related to the convective/diffusive transport of water.*

 We have changed the text in this section to clarify this point:

 “…wherein the materials that are highly effective at P removal generally s **how** poor **performance for** water **transport.**”

*6.  The authors mention new developments in the membrane design/materials field. It might be important to also comment on the cost and scalability of these new membrane designs, as these two factors are often extremely limiting for new membrane materials. For researchers in this area reading this article, it’s important for people to think about cost and scalability from the beginning when designing new materials for this application.*

 We agree with the Reviewer that cost and scalability of new membrane materials are very important to consider from the earliest stages of research. We also think that there are some membrane technologies using off-the-shelf membranes (e.g. electrodialysis) that are more well-established and could be adapted for use with NSSS potentially without the need for development of new membranes. We have added the following to this section to clarify this point:

 “ **However, cost and scalability of any new membrane materials must be considered from the earliest stages of development for application in NSSS, particularly for deployment in low- and middle-income countries. Opportunities also exist in modification of existing membranes and processes to tailor them for use in NSSS.**”

# Peer review: Reviewer response for version 1

**DOI:** 10.21956/gatesopenres.14033.r27000

**Published:** 2019-04-11

**Authors:** Kara L. Nelson

**Affiliations:** 1 Department of Civil and Environmental Engineering, University of California, Berkeley, Berkeley, CA, USA

The authors address an important challenge for emerging sanitation technologies, which is the removal of nitrogen and phosphorus. A new international standard requires certain removals, and yet mature technologies that work at small scales do not yet exist. Overall the review is insightful and should help to inspire further innovation. However, more attention is needed to some topics:

  

1. The authors start with the premise that the N and P removals required by ISO 30500 make sense. Do the authors agree that this requirement makes sense in all scenarios?  
2. The authors don’t discuss that NSSS can produce different types of waste streams, depending on whether water is used for flushing, and whether urine and feces are separated. Also, some NSSS treatment technologies produce concentrated waste streams that need further treatment (N and P removal). It would be helpful if the authors can briefly summarize these different streams that can be targeted for N and P removal.
3. Table 1: Caption states that the table provides a summary of N and P removal methods, but some of the methods only work for N (air stripping and breakpoint chlorination), and chemical precipitation is primarily developed for P (via struvite, which only removes about 10% of N). These are crucial distinctions. The Table should be revised to indicate which approaches work for N, P, or both.
4. Breakpoint chlorination section: Although stated in Table 1, it is worth mentioning in the text that this method does not allow recovery of N in a usable form (only removal as N2).
5. Chemical precipitation section: This section makes it seem as if all precipitation strategies are similar in terms of their removal levels of N and P, but in fact the removal potential completely depends on the stoichiometry of the precipitate that is produced. These distinctions should be made.  
6. Ion exchange section: Is there any literature that can be cited for Polonite?  
7. Algal-based systems: These will require large outdoor areal footprints to provide algae with sufficient sunlight. This limitation should be mentioned.

Does the article adequately reference differing views and opinions?

Partly

Are all factual statements correct, and are statements and arguments made adequately supported by citations?

Partly

Is the Open Letter written in accessible language?

Yes

Where applicable, are recommendations and next steps explained clearly for others to follow?

Yes

Is the rationale for the Open Letter provided in sufficient detail?

Yes

Reviewer Expertise:

Treatment technologies for wastewater, urine, fecal sludge, drinking water, water reuse.

I confirm that I have read this submission and believe that I have an appropriate level of expertise to state that I do not consider it to be of an acceptable scientific standard, for reasons outlined above.

# Response

**Published:** 2020-05-08

**Authors:** Lena Trotochaud

We sincerely thank the Reviewer for their insightful comments and suggestions. We have addressed each of their comments below, and we feel that the manuscript is much improved due to their feedback. The Reviewer's comments are copied below in *italic text*, and our responses are in plain text. Changes to the Manuscript are shown in **bold text.**

*The authors address an important challenge for emerging sanitation technologies, which is the removal of nitrogen and phosphorus. A new international standard requires certain removals, and yet mature technologies that work at small scales do not yet exist. Overall the review is insightful and should help to inspire further innovation. However, more attention is needed to some topics:*

 We sincerely thank the Reviewer for their positive comments regarding the manuscript and for the helpful suggestions for improvement.

*1.  The authors start with the premise that the N and P removals required by ISO 30500 make sense. Do the authors agree that this requirement makes sense in all scenarios?*

 The Reviewer raises an excellent point. However, we point out that we make no claims regarding the suitability nor the sensibility of the ISO 30500 standards; the fact is that these standards exist and are likely to be applied to at least some of the RTTC systems. We think that the appropriateness of the ISO 30500 standards is an important conversation that researchers and policy makers in this field should continue to have. However, we believe that it is not within the scope of the current work to opine on this issue.

 To add a bit of commentary: One overarching conclusion from our Open Letter is that a one-size-fits-all approach to NSSS is not likely to be successful. One could extrapolate then also that a one-size-fits-all approach to regulation of NSSS may be inappropriate. While one could argue that certain metrics (e.g. pathogen reduction) are non-negotiable due to the immediate implications for human health, blanket requirements for N and P removal may be inappropriate or restrictive in certain cases. For example, what if a certain NSSS produces pathogen-free, liquid effluent that contains ammonia and phosphate in concentrations appropriate for direct application as a fertigation solution? Does it makes sense to require that the N and P be removed, for example by precipitation, only for that solid product to be re-dissolved later in the same reclaimed water at a later time point for fertigation purposes? Ultimately, the ISO 30500 standards have already been adopted in several countries (including Senegal, the USA, and Canada) and adoption is expected to continue, so the sensibility of the standards may already be a moot point (unless there are future opportunities for revision of the standards).

*2.  The authors don’t discuss that NSSS can produce different types of waste streams, depending on whether water is used for flushing, and whether urine and feces are separated. Also, some NSSS treatment technologies produce concentrated waste streams that need further treatment (N and P removal). It would be helpful if the authors can briefly summarize these different streams that can be targeted for N and P removal.*

 We thank the reviewer for this comment, which is related to concerns raised also by Reviewer 1. The ISO 30500 standards specifically dictate removal from N and P from *liquid* effluent; we have made changes at several points in the manuscript to clarify this limit in scope. (Please see the response to Reviewer 1 for details.)

 We have also added text in the introduction to address the point of different types of waste streams:

 “ **The technological approach for N and P removal from liquid effluent will depend to a great extent on whether, how, and when urine and feces are separated from each other. Also important is whether and to what extent urine is diluted by water used for toilet flushing (and personal washing, where applicable).**”

*3.  Table 1: Caption states that the table provides a summary of N and P removal methods, but some of the methods only work for N (air stripping and breakpoint chlorination), and chemical precipitation is primarily developed for P (via struvite, which only removes about 10% of N). These are crucial distinctions. The Table should be revised to indicate which approaches work for N, P, or both.*

 We have added a column to the table indicating which approaches work for N, P, or both.

*4.  Breakpoint chlorination section: Although stated in Table 1, it is worth mentioning in the text that this method does not allow recovery of N in a usable form (only removal as N2).*

**

 We have added item 6 to the list of “additional issues with breakpoint chlorination”:

 “… **(6) does not allow for recovery of N in a bioavailable form.**”

*5.  Chemical precipitation section: This section makes it seem as if all precipitation strategies are similar in terms of their removal levels of N and P, but in fact the removal potential completely depends on the stoichiometry of the precipitate that is produced. These distinctions should be made.*

 We thank the Reviewer for this comment, which is also similar to concerns raised by Reviewer 1. We have added a discussion and several references related to the nuances of chemical precipitation. We have also clarified in Table 1 which cations are appropriate for only P removal vs both N and P removal together.

 “ **The choice of cation used for precipitation is critical and affects myriad factors including overall cost, P removal efficacy, optimal pH and temperature required for P removal, and reusability/bioavailability of the P-containing precipitate.[Melia 2017] Resulting trade-offs need to be carefully considered depending on the context and effluent requirements. For example, the Fe and Al salts used for P precipitation are typically much less expensive than Ca and Mg salts; however, Fe and Al phosphate precipitates are not suitable for direct use as fertilizers due to low P bioavailability.[Desmidt, 2014] On the other hand, using Mg for struvite precipitation can enable simultaneous removal of phosphate and ammonium in a 1:1 stoichiometric ratio and has been shown to be widely bioavailable;[Melia 2017] however, struvite precipitation has a narrow window of optimal pH and cannot be relied upon as the sole method of N removal due to the much higher concentrations of ammonia/ammonium relative to phosphate.[Mehta, 2015] Furthermore, more research is required to address concerns regarding the presence of contaminants in precipitates where fertilizer use is intended. [Desmidt 2014, Mehta 2015, Melia 2017]**”

*6.  Ion exchange section: Is there any literature that can be cited for Polonite?*

 We thank the reviewer for pointing out this oversight. We have added the following references which describe testing of Polonite:

 Brogowski A, Renman G: Characterization of opoka as a basis for its use in wastewater treatment. *Pol. J. Environ. Stud.* 2004;13(1):15-20.

 Gustafsson JP, Renman A, Renman G, Poll K: Phosphate removal by mineral-based sorbents used in filters for small-scale wastewater treatment. *Water Res.* 2008;42(1-2):189-97. 10.1016/j.watres.2007.06.058

 Renman A, Renman G: Long-term phosphate removal by the calcium-silicate material Polonite in wastewater filtration systems. *Chemosphere*. 2010;79(6):659-64. 10.1016/j.chemosphere.2010.02.035

 Cucarella V, Zaleski T, Mazurek R, Renman G: Effect of reactive substrates used for the removal of phosphorus from wastewater on the fertility of acid soils. *Bioresour. Technol.* 2008;99(10):4308-14. 10.1016/j.biortech.2007.08.037

 We have also re-arranged parts of this section to improve the flow of the narrative, and added a section describing work on hybrid anion-exchange (HAIX) materials for phosphate removal, in part as a response to a query raised by Reviewer 3:

 “ **Hybrid anion-exchange resins (HAIX) consist of iron (hydr)oxide particles embedded within anion-exchange polymers and have been tested with a variety of wastewater streams, including source-separated urine.[Sengupta 2011, O’Neal 2013] The operational capacity of HAIX media depends on both the influent P concentration and the target effluent P concentration,[Martin 2017, O’Neal 2013] and thus use of HAIX in NSSS will need to be assessed on a case-by-case basis. More work is also needed to demonstrate the economic feasibility of these materials (including the regeneration process) for use in small-scale NSSS.**”

 Sengupta S, Pandit A: Selective removal of phosphorus from wastewater combined with its recovery as a solid-phase fertilizer. *Water Res.* 2011;45(11):3318-30. 10.1016/j.watres.2011.03.044

 O’Neal JA, Boyer TH: Phosphate recovery using hybrid anion exchange: Applications to source-separated urine and combined wastewater streams. *Water Res.* 2013;47(14):5003-17. 10.1016/j.watres.2013.05.037

 Martin BD, De Kock L, Gallot M, Guery E, Stanowski S, MacAdam J, McAdam EJ, Parsons SA, Jefferson B: Quantifying the performance of a hybrid anion exhcnager/adsorbent for phosphorus removal using mass spectrometry coupled with batch kinetic trials. *Environ. Technol.* 2018;39(18):2304-14. 10.1080/09593330.2017.1354076

*7.  Algal-based systems: These will require large outdoor areal footprints to provide algae with sufficient sunlight. This limitation should be mentioned.*

 We have added the following text to the section on algal-based systems:

 “ **In particular, adapting algal-based systems to optimize light exposure in a minimal areal footprint would be critical for implementation in high population-density areas.**”

# Peer review: Reviewer response for version 1

**DOI:** 10.21956/gatesopenres.14033.r26951

**Published:** 2019-03-13

**Authors:** Susan K. De Long

**Affiliations:** 1 Department of Civil and Environmental Engineering, Colorado State University, Fort Collins, CO, USA

**General comments:**

 This article presents a very useful and timely review of non-biological methods for removing N&P from wastewater that are suitable for small-scale systems. Generally I found the review well organized and well-written, and the presented content is highly practical. My overarching suggestions are that I would have liked to see a larger focus on opportunities for nutrient reuse throughout the manuscript text, and additionally the authors should do a better job of delineating which technologies are for N vs P in Table 1 and in the text. Additionally, the various forms of N and P should be more clearly indicated (e.g. inorganic vs. organic forms). My only other suggestions relate to adding more detail and clarification on some points, which I have listed below.

**Specific comments:**

1. I think it’s worth briefly explaining the ISO 30500 standards in the introduction, at their first mention. Not all readers will be familiar, and this document is actually quite broadly useful beyond NSSS.
2. The design of Table 1 is not optimal. The use of italic text is very subtle. I would suggest a different and more obvious notation for indicating the limitations for use in NSSS. Also, by the title and text it is really not clear which methods are for N and with are for P. I suggest the applicability for each nutrient be separately indicated.
3. I would have liked to see text specifically discussing the column in Table 1 dealing with nutrient recovery. Ability to reuse nutrients should be considered as a critical criterion for technology development and selection. Although reuse possibilities are summarized in the table, mentioning them in the text would bring more attention to the importance of this issue.
4. Breakpoint chlorination is said to release “almost no nitrous oxide”. It would be highly preferred to be more quantitative in this statement given the potency of N _2_0 as a GHG.
5. For breakpoint chlorination, a reference should be added related to byproducts which are toxic and carcinogenic to direct the reader to detailed information.
6. Please explain why breakpoint chlorination leads to high TDS in the effluent.
7. The chemical precipitation section should be expanded to individually discuss the different metals added. Please add text explaining how the choice of Fe vs. Al vs. Ca vs. Mg affects efficiency and whether the end product is reusable. Struvite is mentioned, but nothing is said about the other precipitates.
8. For the ion exchange section, change “rate” to “efficiency”. 95% is not a rate.
9. For “Reconsidering biological N and P remediation at small scales”, the meaning of “native” for biofilms is not specified. Some of the transitions in this section are rough as well.
10. Please clarify that for inorganic P capture with FeCl3, reuse is not viable. Please also more clearly discuss inorganic vs organic forms of P.

Does the article adequately reference differing views and opinions?

Yes

Are all factual statements correct, and are statements and arguments made adequately supported by citations?

Partly

Is the Open Letter written in accessible language?

Yes

Where applicable, are recommendations and next steps explained clearly for others to follow?

Partly

Is the rationale for the Open Letter provided in sufficient detail?

Yes

Reviewer Expertise:

Environmental engineering, biological treatment, applied microbiology, contaminant biodegradation, nutrient recovery

I confirm that I have read this submission and believe that I have an appropriate level of expertise to confirm that it is of an acceptable scientific standard.

# Response

**Published:** 2020-05-08

**Authors:** Lena Trotochaud

We sincerely thank the Reviewer for their insightful comments and suggestions. We have addressed each of their comments below, and we feel that the manuscript is much improved due to their feedback. The Reviewer's comments are copied below in *italic text*, and our responses are in plain text. Changes to the Manuscript are shown in **bold text.**

*This article presents a very useful and timely review of non-biological methods for removing N&P from wastewater that are suitable for small-scale systems. Generally I found the review well organized and well-written, and the presented content is highly practical. My overarching suggestions are that I would have liked to see a larger focus on opportunities for nutrient reuse throughout the manuscript text, and additionally the authors should do a better job of delineating which technologies are for N vs P in Table 1 and in the text. Additionally, the various forms of N and P should be more clearly indicated (e.g. inorganic vs. organic forms). My only other suggestions relate to adding more detail and clarification on some points, which I have listed below.*

 We sincerely thank the Reviewer for their positive comments regarding the manuscript and for the helpful suggestions for improvement.

 On the point of the Reviewer’s desire to “see a larger focus on opportunities for nutrient reuse throughout”, we agree that nutrient reuse is indeed a critically important topic in addition to nutrient removal. However, the scope of this article was specifically limited to analysis of nutrient *removal*, as this is the metric specified in the ISO 30500 standard for water reuse.

 There are many questions that surround nutrient reuse that are extremely case specific (e.g. Is the reuse for landscaping, agriculture, or livestock nutritional supplement?; For agricultural use, what are the specific crops to be cultivated and does the method of nutrient recovery support this?; What additional costs/added chemicals/processes are involved for nutrient reuse and are these affordable in a low- and/or middle-income country context?; Is the reuse product safe in terms of pathogen/heavy metals/trace pharmaceuticals content?)  We believe that an adequate treatment of nutrient *reuse* is therefore outside the scope of the current article and would best be reviewed separately. We have added the following text (emphasis included) to the main text to indicate this limitation in scope while acknowledging the importance of nutrient reuse:

 “ **It is important to note that the ISO 30500 standard is written with specific guidance (including liquid effluent reduction threshold values) for *removal* of N and P. While *recovery* and *reuse* of nutrients are critical topics for sustainability and implementation of NSSS technologies, the primary technological hurdle currently facing RT system deployment and water reuse is nutrient *removal*. We briefly mention situations where recovery/reuse is possible and indicate this also in Table 1, as we feel this is an important and exciting area for continued research. However, a comprehensive review of nutrient recovery/reuse is outside the scope of this Open Letter.**”   

 On the point of “delineating which technologies are for N and P in Table 1 and in the text”, we received comments from all Reviewers regarding the formatting of Table 1 and have made several changes in the revised version. A new column has been added which indicates whether the technologies mentioned are appropriate for removal of N, P, or both. Additionally, we have changed the formatting of some values, including shading of certain cells, to more clearly delineate differences. (Note – our original version of this table included different colors of text, but we were informed by the editorial staff that the use of colored text is not allowed.)

 On the point of more clearly indicating the organic and inorganic forms of N and P, we thank the Reviewer for pointing out this ambiguity. The term “nutrients” or “N and P” are used frequently in the literature (including in the original version of our manuscript), but are not sufficiently precise when discussing the chemistry required for removal of organic vs inorganic N and P components. The discussion of organic vs inorganic fractions of N and P in human excreta has been covered in an extensive survey of the literature by Rose et al. (2015); specifically, 90% of total N and 50-65% of total P is contained in the urine fraction. More specifically, most of the organic fractions of N and P are excreted in feces, and thus the inorganic N and P (primarily ammonia from hydrolyzed urine and orthophosphate) is the fraction with the highest contribution to the liquid effluent. Ammonia and orthophosphate are also the nutrients of highest concern with regard to anthropogenic nutrient pollution. We have added the following text to the abstract and introduction to clarify this point:

 In the abstract:  “…emerging technologies for N and P **(specifically ammonia/ammonium and orthophosphate)** removal…”

 In the introduction, paragraph 1: “In particular, algal blooms, caused by eutrophication due to high levels of nitrogen and phosphorus (N and P **, primarily as ammonia/ammonium and orthophosphate**),…”

 In the introduction, after paragraph 2: “ **The technological approach for N and P removal from liquid effluent will depend to a great extent on whether, how, and when urine and feces are separated from each other. Also important is whether and to what extent urine is diluted by water used for toilet flushing (and personal washing, where applicable). Urine contains approximately 90% of the total N and 50-65% of the total P in human excreta, and the chemical components are largely inorganic compounds (assuming urea hydrolysis); in contrast, the contributions to N and P from feces are largely organic (proteins and bacterial biomass). [Rose et al., 2015] For the purposes of this Open Letter, we focus on removal of ammonia/ammonium and orthophosphate, which are the majority water-soluble contributors to N and P in human excreta and the compounds of primary concern for anthropogenic nutrient pollution.”**

 The introduction of the above text requires an additional citation to the manuscript:

 Rose C, Parker A, Jefferson B, et al.: The characterization of feces and urine: A review of the literature to inform advanced treatment technology. *Crit Rev Environ Sci Tec.* 2015;45(17):1827-79. 10.1080/10643389.2014.1000761

 The Reviewer’s suggestion to clarify that ISO 30500 refers specifically to removal of N and P from *liquid* effluent also clarifies that the water soluble fractions of N and P are most relevant for this discussion. We have further clarified that that we are focused on liquid effluent:

 In the abstract: “ However, increasingly stringent **liquid** effluent standards for N and P…”

 In the introduction, paragraph 2: “ **Treatment of the liquid fraction of NSSS waste to enable non-potable water reuse (e.g. for toilet flushing) has been a focus of many reinvented toilet (RT) technologies and guides the scope of this Open Letter.**”

 and paragraph 3: “…ISO 30500 requires 70% and 80% reductions in total N and total P, respectively, in NSSS **liquid** effluent.”

***Specific comments:***

*1.  I think it’s worth briefly explaining the ISO 30500 standards in the introduction, at their first mention. Not all readers will be familiar, and this document is actually quite broadly useful beyond NSSS.*

 We thank the Reviewer for this suggestion. We have found it particularly helpful for clarifying some of the other points brought up by all three reviewers, in that the ISO 30500 specifically dictates nutrient removal requirements from *liquid effluent*, and this was not explicitly clear in the original version of our manuscript and indeed may not have been clear to readers who are unfamiliar with the standard. We have added the following to the introduction, paragraph 3, after the first mention of ISO 30500:

 “ **The ISO 30500 standards provide guidance for safe onsite treatment of human excreta and non-potable water reuse, and includes threshold performance metrics for liquid effluent quality, including chemical oxygen demand (COD), total suspended solids (TSS), nutrients (N and P), and specific pathogens.**”

*2.  The design of Table 1 is not optimal. The use of italic text is very subtle. I would suggest a different and more obvious notation for indicating the limitations for use in NSSS. Also, by the title and text it is really not clear which methods are for N and with are for P. I suggest the applicability for each nutrient be separately indicated.*

 We thank the reviewer for this comment; indeed, all three Reviewers suggested improvements to the formatting and content of Table 1. We have updated the table in the new version and feel that the changes address the concerns of all Reviewers.

*3.  I would have liked to see text specifically discussing the column in Table 1 dealing with nutrient recovery. Ability to reuse nutrients should be considered as a critical criterion for technology development and selection. Although reuse possibilities are summarized in the table, mentioning them in the text would bring more attention to the importance of this issue.*

 We agree with the Reviewer that nutrient recovery should be considered as critical. However, the ISO 30500 specifically provides guidance for nutrient *removal* requirements and only briefly mentions nutrient *recovery*. We believe that the complex and multifaceted issues of nutrient recovery/reuse are outside the scope of the current work, and we have updated the language in the main text to clearly delineate the scope of this letter.

*4.  Breakpoint chlorination is said to release “almost no nitrous oxide”. It would be highly preferred to be more quantitative in this statement given the potency of N _2_0 as a GHG.*

 It is possible in principle to form N _2_O from decomposition of chloramines without an excess of chlorine, however in practice, N _2_O is not observed as a byproduct of breakpoint chlorination due to the large excess of chlorine that is present. The text has been edited and a citation added to clarify this point:

 “with no **measurable** nitrous oxide formation **under appropriate operating conditions** ^20^. **[Pressley, 1972]**”

 Pressley TA, Bishop DF, Roan SG: Ammonia-nitrogen removal by breakpoint chlorination. *Environ. Sci. Technol.* 1972;6(7):622-8. 10.1021/es60066a006

*5.  For breakpoint chlorination, a reference should be added related to byproducts which are toxic and carcinogenic to direct the reader to detailed information.*

 Byproducts of breakpoint chlorination are typically the same as for other chlorine disinfection methods, e.g. chloramines, as described later in the paragraph in question. We have also added three references which specifically discuss breakpoint chlorination byproducts:

 “…undesirable oxidation byproducts, many of which are toxic or carcinogenic. **[Yang 2005; Shah 2012; How 2017]**”

 Yang X, Shang C, Huang J-C: DBP formation in breakpoint chlorination of wastewater. *Water Res.* 2005;39(19):4755-67. 10.1016/j.watres.2005.08.033

 Shah AD, Mitch WA: Halonitroalkanes, halonitriles, haloamides, and N-nitrosamines: A critical review of nitrogenous disinfection byproduct formation pathways. *Environ. Sci. Technol.* 2012;46(1):119-31. 10.1021/es203312s

 How ZT, Kristiana I, Busetti F, Linge KL, Joll CA: Organic chloramines in chlorine-based disinfected water systems: A critical review. *J. Environ. Sci.* 2017;58:2-18. 10.1016/j.jes.2017.05.025

*6.  Please explain why breakpoint chlorination leads to high TDS in the effluent.*

 We have edited the text to clarify this point:

 “…(2) **need for chemical addition to neutralize pH, causing** large increases in total dissolved solids in the treated effluent…”

*7.  The chemical precipitation section should be expanded to individually discuss the different metals added. Please add text explaining how the choice of Fe vs. Al vs. Ca vs. Mg affects efficiency and whether the end product is reusable. Struvite is mentioned, but nothing is said about the other precipitates.*

 We have added to the text in this section to briefly describe the use of different metals for P removal and the trade-offs associated with each; the topic has been extensively reviewed elsewhere, and we have also added some relevant references.

 “ **The choice of cation used for precipitation is critical and affects myriad factors including overall cost, P removal efficacy, optimal pH and temperature required for P removal, and reusability/bioavailability of the P-containing precipitate.[Melia 2017] Resulting trade-offs need to be carefully considered depending on the context and effluent requirements. For example, the Fe and Al salts used for P precipitation are typically much less expensive than Ca and Mg salts; however, Fe and Al phosphate precipitates are not suitable for direct use as fertilizers due to low P bioavailability.[Desmidt, 2014] On the other hand, using Mg for struvite precipitation can enable simultaneous removal of phosphate and ammonium in a 1:1 stoichiometric ratio and has been shown to be widely bioavailable;[Melia 2017] however, struvite precipitation has a narrow window of optimal pH and cannot be relied upon as the sole method of N removal due to the much higher concentrations of ammonia/ammonium relative to phosphate.[Mehta, 2015] Furthermore, more research is required to address concerns regarding the presence of contaminants in precipitates where fertilizer use is intended.[Desmidt 2014, Mehta, 2015, Melia 2017]**”

 Melia PM, Cundy AB, Sohi SP, Hooda PS, Busquets R: Trends in the recovery of phosphorus in bioavailable forms from wastewater. *Chemosphere* 2017;186:381-95. 10.1016/j.chemosphere.2017.07.089

 Desmidt E, Ghyselbrecht K, Zhang Y, Pinoy L, Van der Bruggen B, Verstraete W, Rabaey K, Meesschaert B: Global phosphorus scarcity and full-scale P-recovery techniques: A review. *Crit. Rev. Environ. Sci. Tec.* 2015;45(4):336-84. 10.1080/10643389.2013.866531

 Mehta CM, Khunjar WO, Nguyen V, Tait S, Batstone DJ: Technologies to recover nutrients from waste streams: A critical review. *Crit. Rev. Environ. Sci. Tec.* 2015;45(4):385-427. 10.1080/10643389.2013.866621

*8.  For the ion exchange section, change “rate” to “efficiency”. 95% is not a rate.*

 This change has been made.

*9.  For “Reconsidering biological N and P remediation at small scales”, the meaning of “native” for biofilms is not specified. Some of the transitions in this section are rough as well.*

 We have added a sentence to more clearly define what we mean by “native” biofilms. Additional small changes in this paragraph have been made to help with transitions.

 “ **The lines between traditional “biological” treatment processes (i.e. systems intentionally inoculated with specific microbial strains) and truly “non-biological” treatment methods are likely already blurred in many RT systems due to the high microbial load of feces, which could yield formation of “native” biofilms on or within system components.** The role of native biofilms in nutrient sequestration **thus** cannot be ignored, and the conditions which favor biological N and P sequestration in **native** biofilms could be optimized; more work is needed in this area, and would be specific to a given wastewater stream **and treatment context**.”

*10.  Please clarify that for inorganic P capture with FeCl3, reuse is not viable. Please also more clearly discuss inorganic vs organic forms of P.*

 We have already described clarifications to the introduction to limit the scope of this work to orthophosphate. We therefore do not feel it is necessary to clarify the discussion here with regards to inorganic vs organic P.

 The precipitate recovered in the specific case referenced here is vivianite. While the direct bioavailability and use of vivianite is still somewhat controversial, there is promise for its use as a fertilizer precursor or in electronic applications, which was the subject of a review published shortly after submission of our manuscript. We have added the following text and citations:

 “ **(The precipitate recovered in this case was vivianite, which may have value in electronic applications and as a fertilizer precursor.)[Wilfert 2018, Wu 2019]**”

 Wilfert P, Dugulan AI, Goubitz K, Korving L, Witkamp GJ, Van Loosdrecht MCM: Vivianite as the main phosphate mineral in digested sewage sludge and its role for phosphate recovery. *Water Res.* 2018;144:312-21. 10.1016/j.watres.2018.07.020

 Wu Y, Luo J, Zhang Q, Aleem M, Fang F, Xue Z, Cao J: Potentials and challenges of phosphorus recovery as vivianite from wastewater: A review. *Chemosphere*. 2019;226:246-58. 10.1016/j.chemosphere.2019.03.138
